# Enhancing Protease and Amylase Activities in *Bacillus licheniformis* XS-4 for Traditional Soy Sauce Fermentation Using ARTP Mutagenesis

**DOI:** 10.3390/foods12122381

**Published:** 2023-06-15

**Authors:** Andong Zhang, Yudong Ma, Yue Deng, Zhiwei Zhou, Yue Cao, Bin Yang, Jing Bai, Qun Sun

**Affiliations:** 1 Key Laboratory of Bio-Resources and Eco-Environment of the Ministry of the Education, College of Life Sciences, Sichuan University, Chengdu 610064, China; 2 School of China Alcoholic Drinks, Luzhou Vocational and Technical College, Luzhou 646000, China

**Keywords:** *Bacillus licheniformis* mut80, ARTP, protease and amylase, traditional soy sauce, fermentation

## Abstract

This study was conducted to increase the enzymatic activity of *Bacillus licheniformis* XS-4, which was isolated from the traditional fermented mash of Xianshi soy sauce. The mutation was induced by atmospheric and room-temperature plasma (ARTP), and a mutant strain, mut80, was obtained. mut80 exhibited significant increases in protease and amylase activity by 90.54% and 143.10%, respectively, and the enhanced enzymatic activities were stably maintained after 20 consecutive incubations. Re-sequencing analysis of mut80 revealed that the mutation sites were located in 1518447(AT-T) and 4253106(G-A) in its genome, which was involved in the metabolic pathways of amino acids. The expression of the protease synthetic gene (*aprX*) increased 1.54 times, while that of the amylase gene (*amyA*) increased 11.26 times, as confirmed via RT-qPCR. Using ARTP mutagenesis, the present study proposes a highly efficient microbial resource with enhanced protease and amylase activity provided by *B. licheniformis*, which can potentially be used to improve the efficiency of traditional soy sauce fermentation.

## 1. Introduction

The microbial community structure in traditional fermented mash changes significantly with fermentations [1,2]. In Xianshi Town, Luzhou City, and Sichuan Province, *Bacillus* spp. is the dominant genus, accounting for 67% of the microbial community in soy sauce grains [3]. Many studies have proven that *Bacillus* spp. can metabolize sauce flavor and aroma substances [2]. For example, when *Bacillus subtilis* CS1.03 was used for fermentation in soy sauce, the ammoniacal nitrogen was 10.7 g/L, and the contents of phenol, aldehyde, and ketone in the soy sauce were all increased, which could significantly improve the flavor of the soy sauce [4,5]. *B. amyloliquefaciens* JY06 can be used to eliminate ethyl carbonate and enhance the flavor of soy sauce [6], and *B. amyloliquefaciens* JY06 can reduce the ‘browning’ reaction during the production of soy sauce [6]. *Bacillus subtilis* natto breaks down the proteins in natto and increases the diversity of amino acid compounds [7]. We found that *Bacillus* spp. forms a large number of enzymes, which can promote the Maillard reaction and give fermented products a special flavor [8,9,10]. For example, *Bacillus* SQVG18 and SQVG22 exhibit high protease, cellulase, and phytase activity, which can increase the content of lysine and methionine in the re-fermentation process [11]. *Bacillus* species such as *Bacillus subtilis* and *Bacillus amyloliquefaciens* are widely used to produce fermented foods from soybeans and locust beans in Asian and West African countries [12]. The above studies confirm that *Bacillus* is a potential bacterial genus in Chinese traditional fermented mash. However, the natural strains have a longer fermentation time due to their lower enzymatic activity. The traditional fermentation of soy sauce takes 3–5 years [13]. Thus, there is a need to find new strains to improve fermentation efficiency and quality [3,14,15]. Therefore, obtaining a stable mutant strain with increased enzyme activity is crucial.

To improve the robustness of microbial strains with target traits, various traditional, classical, and advanced strain-improvement techniques have been explored for decades [16,17]. Atmospheric and room-temperature plasma (ARTP) is a novel and promising physical mutagenesis technique that utilizes a plasma jet generated at atmospheric pressure and room temperature to induce mutations in various microorganisms. The plasma jet contains various reactive species, including charged particles, free radicals, excited neutral substances, and ultraviolet radiation, which can cause DNA damage and induce mutations in microorganisms [18,19]. ARTP does not require a vacuum system and can be operated under atmospheric pressure, which reduces the cost and complexity of the equipment. ARTP can induce a high frequency of mutations, which increases the probability of obtaining desirable mutants.

Atmospheric and room-temperature plasma (ARTP) has emerged as a promising technique in microbiology. It has found extensive applications in various areas, such as the mutation breeding of industrial microorganisms, improving the fermentation performance of yeasts, enhancing the antimicrobial activity of probiotics, and the production of bioactive compounds. ARTP has proven to be a valuable tool for inducing mutations in microorganisms, leading to the development of novel strains with improved traits. In addition, it has been shown to be an effective method for enhancing the production of bioactive compounds, with potential applications in the pharmaceutical and food industries. Overall, ARTP has shown great potential and versatility in microbiology, making it a valuable tool for microbial biotechnology research [20,21]. For example, *Myceliophthora thermophila*’s superoxide dismutase activity increased by 221.13%, and polyphenol oxidase activity increased by 486.04%, after ARTP mutation [14]. A *Streptomyces hiroshimensis* SK43.001 with transphosphatidylation activity was mutated by ARTP, and the enzyme activity of mutant Sk43.001-11 was increased by 82% [22]. Therefore, using ARTP mutagenesis to improve enzyme activity production is a promising approach [16,17,18,19]. It is also an important tool to accelerate the decomposition of raw materials and speed up the brewing process.

The strains in this paper were derived from the fermentation process of soy sauce, which is brewed from soybeans and wheat [23]. The proteins in soybeans are a source of nitrogen for the microorganisms involved in traditional soy sauce fermentation, while the starch in wheat provides a source of carbohydrates. During the fermentation process, the proteins and carbohydrates are broken down into amino acids and sugars, which contribute to the flavor and aroma of the final product [24,25]. The enzymatic breakdown of starch and protein is therefore an important factor in the distinctive flavor of soy sauce. Traditionally, *Aspergillus oryzae* has been the dominant microorganism in the early stages of soy sauce fermentation, and it plays a crucial role in the hydrolysis of both starch and protein [26,27]. *Aspergillus oryzae* produces various enzymes, including amylase and protease, which break down the starch and protein in soybeans and wheat, respectively [28,29,30]. However, during the later stages of soy sauce fermentation, the moisture content of the soy mash decreases, and the salinity increases, which makes it difficult for *Aspergillus oryzae* to grow and continue its enzymatic activity [31,32,33]. As a result, other microorganisms, such as *Bacillus* spp., become dominant during the later stages of fermentation. *Bacillus* spp. is salt-tolerant and adapts well to the harsh fermentation conditions of soy sauce production. It produces various hydrolytic enzymes, including 55 proteases, α-amylase, and β-glucosidase, which continue the breakdown of starch and protein to create the unique flavor and aroma of soy sauce [33,34]. However, while most studies on *Bacillus* spp. have focused on the flavor changes that occur following mixed fermentation, comparatively fewer studies have investigated improving *Bacillus* spp. enzyme activity through mutagenic selection.

Based on the above facts, this study aimed to use ARTP mutagenesis to improve the enzyme activity of *B. licheniformis* XS-4. The wild-type *B. licheniformis* XS-4 was exposed to ARTP mutagenesis, followed by screening to select high-performance mutant strains with higher protease and amylase activity. The mutants were further studied for growth curves, acid tolerance, salt tolerance, thermal tolerance, enzymatic activity, and enzymatic hydrolysis ability. Finally, ARTP mutagenic loci were identified via whole-genome sequencing and re-sequencing analysis, RT-qPCR was conducted to verify the expression of protease and amylase synthesis genes, and RT-qPCR validation was conducted to explore the mechanism of high enzymatic activity in the mutant strain.

## 2. Materials and Methods

### 2.1. Strains and Culture Conditions

The wild strain, *B. licheniformis* XS-4, was isolated from the soy sauce mash via natural sun-brewed fermentation (Xianshi Food Co. Ltd., Luzhou, China). The strain was activated by culturing it with NB solid medium (3.0 g/L yeast extract, 10.0 g/L peptone, 5.0 g/L NaCl, and 20 g/L agar) at 37 °C for 3 days. Then, we selected a single colony, and transferred it to NB liquid medium (3.0 g/L yeast extract, 10.0 g/L peptone, and 5.0 g/L NaCl) at 37 °C, 200 rpm, for 3 days. The cells were collected via centrifugation at 8000 r/min for 2 min, which was used for mutation via ARTP in the next step [23].

### 2.2. ARTP Mutagenesis and Screening

The pellet of *B. licheniformis* XS-4 was re-suspended in phosphate-buffered saline (PBS) at 10^6^ CFU/mL. The suspension (10 μL) was spread on carrier plates for mutation via ARTP-IIIS (Wuxi TMAXTREE Biotechnology Co., Ltd., Wuxi, China) [16,18,20]. The parameter settings were as follows: the radio frequency power input was 120 W, and the treatment time ranged from 0 to 120 s with a time interval of 30 s. After treatment, the cells were washed with distilled water, serially diluted (10^−1^ to 10^−5^), spread on the NB solid medium, and cultured at 37 °C for 3 days. The single colonies were transferred to NB liquid medium, and incubated at 37 °C, 180 rpm, for 2 days. The mutant strains were stored with glycerin at −80 °C [21].

### 2.3. Screening of Mutant Strains with High Protein and Starch Hydrolysis Ability

After the culture was activated, the single colonies of *B. licheniformis* XS-4 and mutants were transferred to NB liquid medium, and incubated at 37 °C, 200 rpm, for 3 days. The hydrolysis capacity of the protein and starch of the mutants were determined using the Oxford cup method [21]. For protein hydrolysis capacity testing, four Oxford cups were placed in NB solid medium supplemented with casein (3.0 g/L yeast extract, 10.0 g/L peptone, 5.0 g/L NaCl, 4 g/L casein, and 20 g/L agar). In two Oxford cups, we injected 100 µL mutants of inoculum, and in the other two, we injected 100 µL XS-4 inoculum as a control. Additionally, they were incubated at 37 °C for 3 days. Similarly, to the protein hydrolysis capacity test, the starch hydrolysis capacity of mutants was measured in the NB solid medium supplemented with soluble starch (3.0 g/L yeast extract, 10.0 g/L peptone, 5.0 g/L NaCl, 2 g/L starch, and 20 g/L agar). The diameter ratio (X) of the hydrolysis circle and strain growing circle was calculated according to Equation (1). When the value of X ≥ 2.500, the strain was considered as having high protease and amylase hydrolysis capacity [24].

(1) $$X=\frac{d_{h}}{d_{s}}$$

where *X* represents the diameter ratio of the hydrolysis circle and strain growing circle; *d_h_* is the diameter of the hydrolysis circle, and *d_s_* is the diameter of the strain growing circle.

### 2.4. The Enzyme Activity Test and the Genetic Stability Analysis of Mut80

According to the results of the screening, the mutant strain mut80 exhibited higher protein and starch hydrolysis capacity than the other mutants; therefore, it was selected for subsequent research. After the culture was activated, the single colonies of XS-4 and mut80 were transferred to 100 mL NB liquid medium at 37 °C, 200 rpm, and incubated for 3 days. After incubation, the supernatant was collected via centrifugation for enzyme activity testing. The acidic protease and α-amylase activities were measured using a Micro Acidic Proteinase Assay Kit and an α-amylase Assay Kit (Solar Bio, Beijing, China). We followed the kit instructions for operation. Furthermore, in order to determine the genetic stability of mut80, it was continuously subcultured for 20 generations. The activities of acidic protease and α-amylase were measured every 5 generations [23].

### 2.5. Whole-Genome Analysis and Resequencing

#### 2.5.1. DNA Extraction

DNA extraction was performed using the Wizard^®^ Genomic DNA Purification Kit (Promega, Madison, WI, USA), whereby DNA quality should meet the conditions of OD260/OD280 = 1.8~2.0, total DNA ≥ 15 μg, and concentration ≥ 50 ng/μL.

#### 2.5.2. Library Construction and Sequencing

The genome was sequenced using a combination of the PacBio RS II Single-Molecule Real-Time Sequencing (SMRT) and Illumina sequencing platforms. After obtaining the sequencing data, the genome size was first determined and evaluated for the presence of plasmids and contamination, and then, the Nanopore sequencing data were corrected using the next-generation sequencing data to reduce the possible error rate in the Nanopore sequencing, thus ensuring that the assembly results had a high confidence level [26].

#### 2.5.3. Genome Assembly, Gene Prediction, and Annotation

The raw data underwent a filtered operation to remove low-quality reads and short read lengths. This was carried out in order to obtain cleaner data with higher quality. The resulting data were assembled into overlapping clusters and transformed into loops to obtain the complete genome data [23]. After assembling the data into overlapping clusters and transforming them into loops, the assembly result was corrected and the starting loci of the loop genome were determined. Basic information in the genome was then predicted. CDS prediction was performed by using the Glimmer platform, plasmid gene prediction by Gene MarkS platform, rRNA prediction by the tRNA scan-SE platform, and rRNA prediction by the Barr nap platform. The specific functional annotation of all predicted coding sequences used sequence comparison tools such as BLAST, combined with the GO, COG, and KEGG databases [26].

#### 2.5.4. Genome Resequencing of Mutagenic Bacteria

BWA software was used to compare the sequenced sequences of mutagenic bacteria with the XS-4 genome of the starting bacteria and to remove sequencing errors due to PCR duplication. The sequencing depth and coverage of the mutant genome relative to the XS-4 genome were determined based on the comparison results, and loci with lower sequencing depth and comparison quality values were excluded from the comparison. Var Scan was used to detect SNPs and small indel-related information in the genome of mutagenic bacteria, and finally, to obtain all mutation loci of mutagenic bacteria relative to the starting XS-4 [26,27].

### 2.6. Biological Characteristic Analysis of Mut80

#### 2.6.1. The Growth Curve Test

After the cultures were activated, the XS-4 and mut80 single colonies were cultured in NB liquid medium at 37 °C, 200 rpm, for 8 h, respectively. Then, the 4% inoculum of XS-4 and mut80 was transferred to a 250 mL conical flask with 100 mL fresh NB liquid medium for incubation at 37 °C, 200 rpm, for 24 h. The absorbance (OD 600 nm) of the inoculum was measured every two hours [28].

#### 2.6.2. Salt Tolerance

After the cultures were activated, the XS-4 and mut80 single colonies were incubated in NB liquid medium under different NaCl concentrations (2%, 4%, 6%, 8%, 10%, 12%, 14%, and 16%). The absorbance of the inoculum was measured at 600 nm after it had been incubated for 24 h at 37 °C [29].

#### 2.6.3. Temperature Tolerance

After the cultures were activated, the XS-4 and mut80 single colonies were incubated in NB liquid medium at different temperature (20 °C, 30 °C, 40 °C, and 50 °C). The absorbance of the inoculum was measured at 600 nm after it had been incubated for 24 h at 37 °C.

#### 2.6.4. pH Tolerance

After the cultures were activated, the XS-4 and mut80 single colonies were incubated in NB liquid medium at different pH levels (3.0, 4.0, 5.0, 6.0, 7.0, 8.0, and 9.0). The absorbance of the inoculum was measured at 600 nm after it had been incubated for 24 h at 37 °C.

### 2.7. Quantitative Real-Time PCR (RT-qPCR)

After activating the culture, the single colonies of XS-4 and the mut80 strain were selected and transferred to fresh NB liquid medium. They were then incubated at 37 °C, 200 rpm, for 24 h. The cells were collected via centrifugation at 4 °C, 10,000 rpm, for 2 min. The total RNA was extracted using the Omega Bacterial Total RNA Extraction Kit (Omega, Norcross, GA, USA) [29]. To obtain cDNA, the total RNA was reverse-transcribed using a Reverse Transcription Kit (Takara, Tokyo, Japan) following the instructions outlined in the manuscript. The primers used in this study are listed in Table S1. The cDNAs were used as templates to perform quantitative real-time PCR, with 16S rRNA as the endogenous control (Tables S2 and S3).

### 2.8. Statistical Analysis

All data are expressed as mean ± standard deviation (SD). Statistical analysis was performed using SPSS Statistics 20.0 and GraphPad Prism 8.0.2. One-way analysis of variance (ANOVA) was performed using Duncan’s multiple comparisons tests with a significance level (*p* < 0.05).

## 3. Results

### 3.1. Whole-Genome Analysis of B. licheniformis XS-4

**Figure 1:**
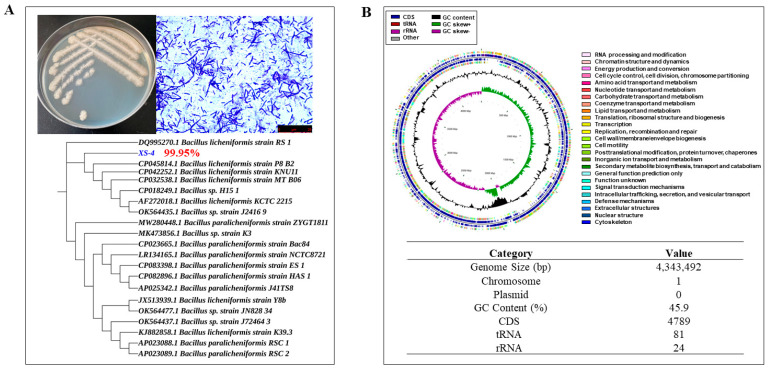
Species identification and the whole-genome analysis of XS-4. (**A**) Identification of XS-4; (**B**) the whole-genome mapping of XS-4.

**Figure 2:**
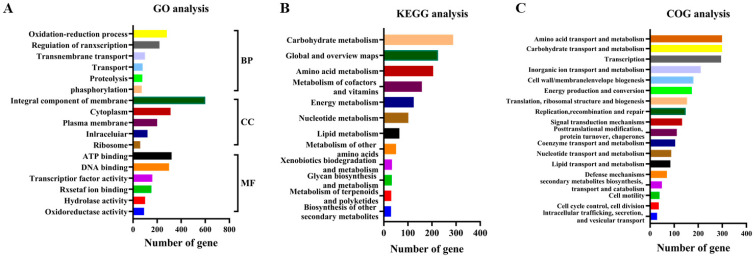
Genome-wide functional annotation of *Bacillus licheniformis* XS-4. (**A**) GO analysis based on the whole-genome annotation; (**B**) KEGG analysis based on the whole-genome annotation; (**C**) COG analysis based on the whole-genome annotation.

The results of the whole-genome analysis determined that the wild strain XS-4 exhibited the highest degree of homology with *B. licheniformis* RS-1, and was therefore named *Bacillus licheniformis* XS-4. The analysis revealed that the genome of XS-4 was composed of a circular chromosome, spanning a size of 4,343,492 base pairs, with a GC content of 45.9%. The genome was found to contain 4789 protein-coding genes (CDS), 81 tRNA genes spanning 20 types, and 24 rRNA manipulators (Figure 1B). Further gene ontology (GO) analysis confirmed that codable genes were most active in the bioregulation of redox and transcriptional processes (Figure 2A). Kyoto Encyclopedia of Genes and Genomes (KEGG) analysis further confirmed that XS-4 was predominantly involved in carbohydrate metabolism and amino acid metabolism (Figure 2B). Furthermore, Clusters of Orthologous Groups (COG) functional annotation revealed that XS-4 had the highest number of genes involved in amino acid transport metabolism, carbohydrate transport metabolism, and transcription (Figure 2C). These three types of functional gene accounted for 8.75%, 8.75%, and 8.63% of all genes, respectively. Through genome-wide prediction and in-depth analysis, XS-4 exhibited important potential in protein and starch hydrolysis, which provided a basis for the further improvement of hydrolysis capacity using ARTP mutagenesis.

### 3.2. ARTP Mutagenesis Enhanced the Casein and Starch Hydrolysis Capacity of XS-4

**Figure 3:**
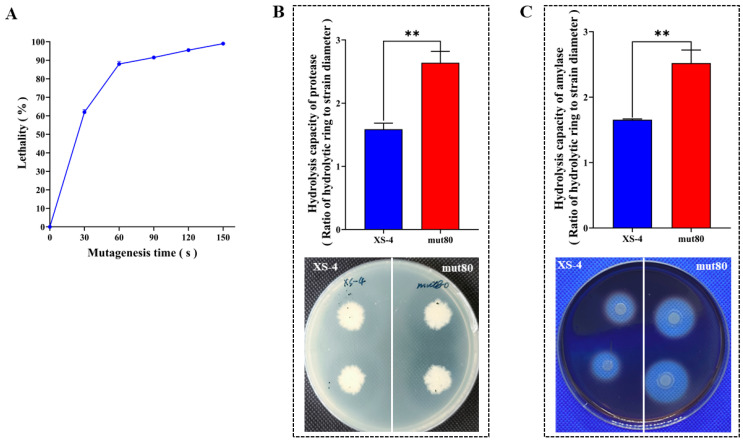
The production capacity of protease and amylase of XS-4 and mut80. (**A**) Lethality rate of ARTP. (**B**) Protease activity of XS-4 and mut80 in hydrolyzing casein. (**C**) Amylase activity of XS-4 and mut80 in hydrolyzing starch. Notes: “**” represents highly significant (*p* ≤ 0.01).

The lethality of XS-4 was 90% at 60 s after ARTP mutagenesis (Figure 3A). The mut80 mutant strain, obtained through ARTP mutagenesis from XS-4, exhibited the largest hydrolysis circle, and was thus selected for further experiments. Compared to XS-4, the hydrolysis capacity of mut80 for casein and starch, as measured using the X value, increased by 66.87% and 52.26%, respectively (Figure 3B). ARTP mutagenesis effectively enhanced the hydrolysis capacity of casein and starch in the mutant.

### 3.3. The Protease and Amylase Activity of Mut80 Could Be Stably Improved

**Figure 4:**
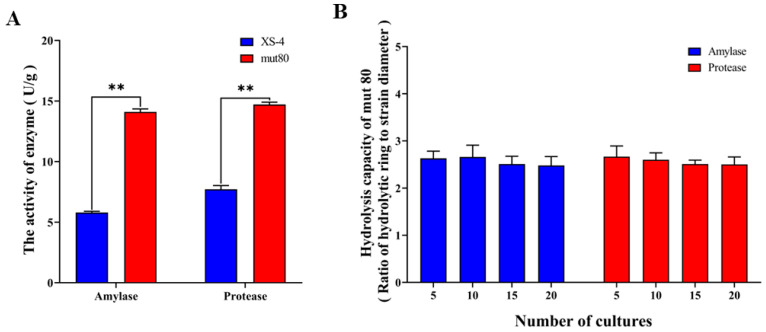
Amylase and protease activity and genetic stability of mut80. (**A**) The amylase and acid protease activity of XS-4 and mut80. (**B**) Determination of the hydrolytic capacity of amylase and protease produced via consecutive culture of mut80. Notes: “**” represents highly significant (*p* ≤ 0.01).

The casein and starch hydrolysis capacity of XS-4 were effectively enhanced by ARTP mutagenesis, so we further explored the changes in the protease and amylase activities of mut80. The acidic protease and amylase activities of mut80 were improved by 90.54% and 143.10% compared to XS-4 (Figure 4A). The genetic stability of strains is the basis for their industrial application. Therefore, we explored the genetic stability of mut80 via successive subculture. There was no significant difference in protease and amylase activities during mut80 subculture from 0 to 20 generations (Figure 4B). In conclusion, ARTP not only significantly improved protease and amylase activities, but also had stable heritability, which exhibited great application potential.

### 3.4. Changes in Biological Characteristic after Mutagenesis

**Figure 5:**
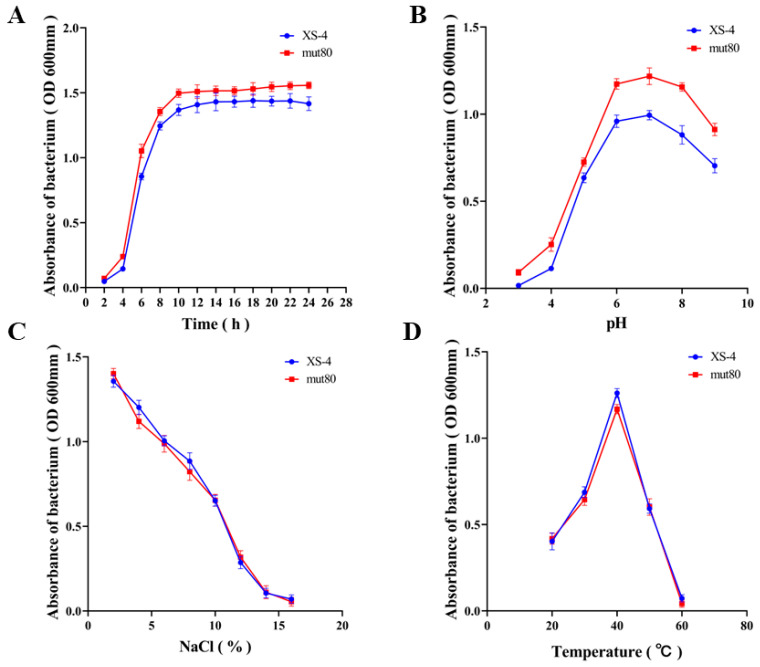
Physiological parameters of mut80. (**A**) Determination of the growth curve of mut80. (**B**) Determination of the acid and alkali tolerance of mut80. (**C**) Determination of salt tolerance of mut80. (**D**) Determination of the temperature tolerance of mut80.

Figure 5A shows the growth curve of XS-4 and mut80, with mut80 exhibiting a slightly faster growth rate than XS-4, and reaching the stationary phase 2 h earlier at 10 h. Furthermore, mut80 could grow in the medium with pH 6 to 8, in which its biomass was higher than that of XS-4 (Figure 5B). The pH tolerance of XS-4 was significantly increased by ARTP mutagenesis. However, there were no significant differences in salt tolerance and thermal tolerance (Figure 5C,D).

### 3.5. Analysis of Resequencing and RT-qPCR

**Figure 6:**
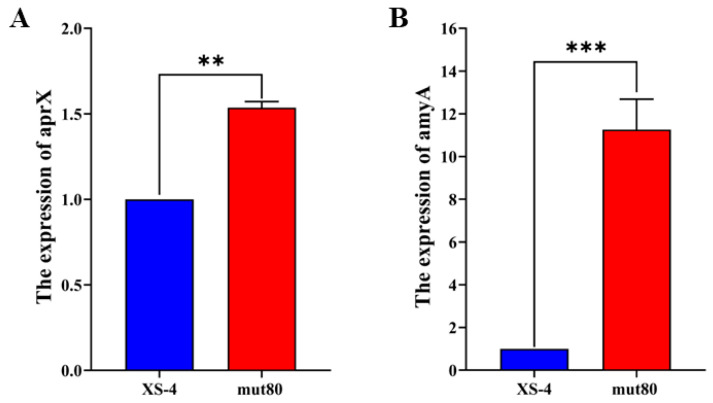
The expression of protease- and amylase-related genes in XS-4 and mut80. (**A**) The mRNA expression of *aprX*. (**B**) The mRNA expression of *amyA*. Notes: “**” represents highly significant (*p* ≤ 0.01), “***” represents highly significant (*p* ≤ 0.001).

In order to understand how ARTP alters protease and amylase activity, we conducted resequencing analysis of mut80, using the whole genome of XS-4 as a reference. We confirmed that ARTP caused effective mutation sites (*ykvZ* and *alsT*) in mut80 (Table 1), with *ykvZ* as a code shift mutation (CT-C) and *alsT* as a point mutation (G-A) (Table 1). *alsT* was annotated via uniport and found to encode a 50.3 kDa hydrophobic protein with many transmembrane structural domains; it was an amino acid carrier protein, a Na^+^/H^+^-dependent alanine carrier, belonging to the AGCS family, mainly involved in the synthesis and metabolism of amino acids [13,30]. *ykvZ* was annotated as an amino acid carrier protein [13,30], with *alsT* and *ykvZ* having important roles in amino acid metabolism. Its inactivation may lead to altered transcript levels in genes encoding related enzyme proteins and amylase genes in metabolic reactions. RT-qPCR analysis of related genes was performed on XS-4 and mut80 cultured for 24 h in terms of their phenotypic differences and resequencing analysis results. As we can see in Figure 6A,B, the acid protease synthesizing gene *aprX* was up-regulated 1.53 times and the amylase synthesizing gene *amyA* was up-regulated 11.26 times.

## 4. Discussion

In our study, we identified a strain of XS-4 with protein and starch hydrolysis ability and found that XS-4 has significant effects on carbohydrate and amino acid signaling pathways via KEGG analysis. The XS-4 strain exhibits notable halotolerance, with the ability to withstand salt concentrations ranging from 10 to 15% while exhibiting optimal growth conditions at a temperature of 40 °C and within a pH range of 6–8. Nonetheless, its inherent enzymatic activity is limited, necessitating further optimization. To this end, we implemented ARTP mutagenesis, yielding a stable mutant strain, mut80, which demonstrated a marked increase in protease and amylase activity by 90.54% and 143.54%, respectively. This enhanced enzymatic potential positions mut80 as a promising candidate for facilitating soy mash fermentation during the mid-to-late stages. Despite the predominance of *Bacillus* in the microbial consortia of traditional soy sauce fermentation, the inherent instability of wild-type strains can result in inconsistent soy sauce quality. In light of this, the enhanced biological activity of the mut80 strain, as obtained in our study, is of great significance. Compared with the wild-type strain XS-4, the salt and heat tolerance of mut80 did not change, but mut80 entered the log phase 2h earlier than XS-4, and the growth of mut80 was increased by 22.54% at pH 7.0. The above data prove that mut80 has an advantage over wild-type strains in fermentation. Moreover, we conducted a preliminary exploration of the mechanisms underlying the enhanced activity of the mut80 enzyme. KEGG analysis further confirmed that XS-4 was predominantly involved in carbohydrate metabolism and amino acid metabolism. It was demonstrated that ARTP resulted in effective mutations in the *ykvZ* and *alsT* genes of amino acid metabolism and that protease-related synthesis genes (*aprX*) were significantly upregulated. It is inferred that the enhanced mut80 protease activity may be caused by the mutation of the genes *ykvZ* and *alsT*. However, ARTP mutagenesis did not cause mutations in the carbohydrate signaling pathway, yet amylase synthesis genes were significantly upregulated. It is inferred that carbohydrate metabolism is influenced by amino acid metabolism, which regulates the expression of amylase. In summary, the present study has comprehensively investigated the molecular mechanisms underlying the enhancement of enzyme activity and the resultant biological properties of the mut80 mutant strain. These findings establish a solid foundation for the potential application of mut80 in traditional soy sauce brewing.

## 5. Conclusions

In the present study, we successfully isolated a strain of XS-4 from the traditional fermented mash of Xianshi soy sauce, which demonstrated a capability to survive in a high-salt environment (10–15%) and at an optimum growth temperature and pH range of 40 °C and 6–8, respectively. However, the enzymatic activity of XS-4 was found to be suboptimal. As a solution, we employed ARTP mutagenesis to obtain a genetically stable and highly active mutant strain, mut80, which exhibited a remarkable increase in protease and amylase enzyme activities by 90.54% and 143.10%, respectively. Importantly, mut80 exhibited stronger growth activity, as well as improved acid and alkali resistance compared to the wild-type strain. Moreover, mut80 demonstrated a genetically stable ability to hydrolyze proteins and starches, indicating its potential as a new strain resource for fermented soy sauce production. Collectively, these findings provide a foundation for further research on the application of mut80 in soy sauce brewing and other related fields.

## Figures and Tables

**Table 1:** Results of mut80 resequencing annotation.

POS	Gene ID	REF	ALT	QUAL	Gene	Uniport	Annotation
1518447	gene1686	CT	C	4955.63	*ykvZ*	D4FVW5	Amino acid carrier protein AlsT
4253106	gene4796	G	A	8498.99	*alsT*	Q45068	H^+^/Na^+^: nitrogen-donor amino acid symporter

## Data Availability

Data is contained within the article.

## Supplementary Materials

The following supporting information can be downloaded at: https://www.mdpi.com/article/10.3390/foods12122381/s1, Table S1: Primers for RT-qPCR; Table S2: Reaction system of RT-qPCR; Table S3: RT-qPCR system.
