# Supplementary material for: Enhancing Protease and Amylase Activities in Bacillus licheniformis XS-4 for Traditional Soy Sauce Fermentation Using ARTP Mutagenesis
Source: Foods. 2023 Jun 15;12(12):2381. doi: 10.3390/foods12122381 (PMC10296987; doi:10.3390/foods12122381)
Supplement: Supplementary file 1 [file foods-12-02381-s001.zip › foods-2385629-supplementary.pdf]

foods

Supplementary materials

Enhancing protease and amylase activities in *Bacillus licheniformis* XS-4 from the traditional soy sauce fermentation by ARTP mutagenesis

Andong Zhang[1], Yudong Ma[1], Yue Deng[2], Zhiwei Zhou[1], Yue Cao[1], Bin Yang[1], Jing Bai[1] and Qun Sun[1*]

1 Key Laboratory of Bio-resources and Eco-environment of the Ministry of the Education, College of Life Sciences, Sichuan University, Chengdu, Sichuan 610064, P. R. China
2 Luzhou Vocational and Technical College, Luzhou 646000, China.
* Correspondence: qunsun@scu.edu.cn; Qun Sun, College of Life Sciences, Sichuan University, 29# Wangjiang Road, Chengdu, Sichuan 610064, P.R. China. Tel: 86-28-8541-8810; Fax: 86-28-8546-048

Supplementary Materials:

Table S1. Primers for RT-qPCR.

Gene	Primers（5′→3′）
aprX	F: AGCCCGTTTTTCGGATAGCA<br>R: GAAACGCCATAAAGGGCAGC
amyA	F: ACGGGGATGTGGTCATCAAC<br>R: GCGCCCCGGAAAATGAAAAT
16S	F: AGAGTTTGATCCTGGCTCAG<br>R: GGTTACCTTGTTACGACTT

Table S2. Reaction system of RT-qPCR.

System	Volume (µl)
TB Green Premix Ex Taq Ⅱ	10.0
ROX Reference Dye Ⅱ	0.4
Primer F	0.6
Primer R	0.6
ddH_2O	7.4
cDNA	1.0

Table S3. RT-qPCR system.

RT-qPCR process	Temperature (°C)	Time (s)
Predegeneration	95	30
40 cycles	95	5
	60	30
	72	20
